# Barriers to Accessing Cervical Cancer Screening and Treatment in the Amazon Region—A Systematic Review

**DOI:** 10.3390/jcm15031206

**Published:** 2026-02-03

**Authors:** Marcia Helena Ribeiro de Oliveira, Sandra Lopes Aparício, José Antônio Cordero da Silva, Domingos Aires Leitão Neto, Sofia B. Nunes, Guilhermina Rêgo

**Affiliations:** 1 Faculty of Medicine, University of Porto, 4200-319 Porto, Portugal; domingos_aires@yahoo.com.br (D.A.L.N.); asnunes@med.up.pt (S.B.N.); 2 RISE-Health, Faculty of Medicine, University of Porto, Alameda Prof. Hernâni Monteiro, 4200-319 Porto, Portugal; smaparicio@med.up.pt (S.L.A.); guilherminarego@med.up.pt (G.R.); 3 Faculty of Medicine, University of the State of Pará, Belém 66087-670, Brazil; corderobel4@gmail.com

**Keywords:** cervical cancer, cervical cancer screening, cervical cancer treatment, barriers to care, barriers to treatment, equity in healthcare access, Amazon Region, developing country

## Abstract

**Background/Objectives**: Unequal access to cervical cancer screening and treatment remains a significant contributor to preventable morbidity and mortality for women in the Amazon Basin, compounded by geographic, social and infrastructural barriers. Given the fragmented nature of the existing evidence, this systematic review aims to synthesize available findings on barriers to cervical cancer screening and treatment for this region. The implications of these findings are examined to inform the development of actionable strategies to improve equity in prevention and care. **Methods**: This systematic review was conducted in accordance with PRISMA 2020. Searches were conducted on 7 November 2025, in PubMed/MEDLINE, Web of Science, and SciELO, utilizing combinations of MeSH terms, keywords, and free-text expressions. Studies were considered eligible if they addressed barriers to cervical cancer screening or treatment among women living in the Amazon Region. Two reviewers independently screened the studies and extracted the relevant data. The risk of bias was assessed using the JBI checklists, the Newcastle–Ottawa Scale, and the MMAT. A narrative synthesis summarized the results. **Results**: Of 57 studies identified, 11 were included. Organizational and health-system barriers were reported most frequently, including scheduling difficulties, long wait times, a shortage of professionals, and equipment unavailability. Socioeconomic barriers were most often related to younger age, low income, limited schooling, and care related expenses. Cultural factors were frequently linked to fear of the procedure and insufficient knowledge about cervical cancer. Geographic barriers included rural residence and travel difficulties. **Conclusions**: This systematic review indicates that disparities in cervical cancer screening in the Amazon region are primarily associated with organizational and health-system-related barriers, together with socioeconomic, cultural, and geographic factors. These findings highlight the need for equitable, multisectoral interventions to strengthen service organization, improve health literacy, and expand timely access to screening and treatment for underserved women.

## 1. Introduction

Worldwide, cancer remains one of the most urgent public health concerns. Projections suggest that, in low and middle income countries, the number of new cancer cases may nearly double by 2040. Cervical cancer (CC) ranks fourth among cancers diagnosed in women [1]. The epidemiology of CC varies considerably across countries and even within national borders, and much of this variation reflects uneven access to screening programs, treatment, and vaccination [2]. In the literature, researchers have found that CC incidence and mortality may reach levels up to eight times higher in low-income settings [3].

The Human papillomavirus (HPV), a known causal factor for CC, is one of the most common sexually transmitted infections, and close to 80% of sexually active people encounter an HPV infection at least once during their lifetime [4]. Preventive measures include screening, HPV vaccination, health education, and healthy lifestyle practices. If detected early, CC’s outcomes are generally favorable, which points to the importance of effective screening programs [5]. World Health Organization guidelines advise achieving a screening coverage of 70% among women aged 35 and older [6]. Modeling studies indicate that routine screening could prevent up to 83% of deaths linked to CC [7].

In resource-limited settings, gaps in primary care and inconsistent screening remain major barriers to effective cancer treatment [8]. Alternative cervical cancer prevention strategies have been proposed to mitigate structural constraints, including limited laboratory capacity, geographic inaccessibility, and challenges related to patient follow-up. Screening methods that provide real-time results, including optoelectronic devices, may complement conventional cytology where laboratory-based testing is limited. Comparative studies indicate that technologies such as TruScreen™ offer acceptable performance, enabling immediate clinical decision-making without the need for sample transport or molecular testing [9].

In parallel, recent evidence has supported more flexible HPV vaccination strategies. High-quality trials have demonstrated that reduced-dose schedules can induce adequate immunogenicity, suggesting the potential to improve vaccine coverage and feasibility in resource-constrained settings. Despite these advances in primary and secondary prevention, equitable access to organized screening and timely treatment remains essential for reducing cervical cancer burden in vulnerable populations [10].

In this review, access is defined as the ability to seek and receive appropriate healthcare when individuals recognize a need for it. It is shaped by a mix of factors, including individual and family circumstances, social and geographic conditions, and the organization and performance of health systems and providers [11]. In many high-income countries, access to quality healthcare is widely regarded as a fundamental right [12].

Existing evidence suggests an association between lower educational attainment and/or income and increased CC risk, especially in populations facing barriers to early detection and treatment [13,14,15]. Additional factors may hinder screening and treatment, including limited knowledge of CC and recommended screening tests, as well as inadequate professional training [3,13,14,15,16].

Limited ease of access to health services reduces utilization and is linked with poorer health outcomes. The impact is evident in cancer care, where treatment usually involves several stages and repeated visits to health facilities [17]. Greater travel demands among cancer patients have been linked to later-stage diagnoses, inadequate treatment, poorer prognosis, and reduced quality of life [18].

The Amazon Region spans over 7 million square kilometers, corresponding to approximately 6% of the Earth’s surface. It is a transboundary biome that spans eight countries encompassing more than 40% of South America’s surface area [19]. Despite its vast resources, this region has historically undergone various human interventions, culminating in complex issues such as low social and economic indicators, poor quality of basic public services, and difficulties in accessing healthcare [20]. This region is not only extensive but culturally diverse, housing Indigenous peoples, riverine populations (“ribeirinhos”), “quilombola” communities, and many other traditional groups [21].

Despite the compounded barriers faced by populations in the Amazon region, the current literature is fragmented, primarily local in scope [22], and a systematic review on the subject has not been conducted. The primary objective of this study is to systematically identify and synthesize the barriers women face in accessing cervical cancer screening and treatment across the Amazon Basin. Based on this synthesis, the review highlights actionable strategies to support policymakers and healthcare providers in promoting equitable cancer prevention and care in the region.

## 2. Materials and Methods

### 2.1. Search Strategy and Selection Process

This systematic literature review followed the PRISMA 2020 Guidelines [23] and was conducted on 7 November 2025. The completed PRISMA 2020 checklist is provided in the Supplementary Materials, in Table S1. The protocol for this review was registered on PROSPERO (CRD420251150753) and on the International Platform of Registered Systematic Review and Meta-analysis Protocols (INPLASY2025120047, doi:10.37766/inplasy2025.12.0047). The research question was: “What are the main barriers that affect access to cervical cancer screening and treatment among women residing in the Amazon Region?”. The PICO components are listed in Table 1. In the SPIDER framework, the components were detailed in Table 2.

A literature search was performed on the following databases: PubMed/MEDLINE, Web of Science, and SciELO. There are no language or date restrictions for each search in the mentioned databases. Other studies were identified through contacting authors or experts and by checking the reference lists (backward citation searching). The search was conducted using a structured combination of MeSH terms, keywords, and free-text terms. All search queries and complete search strategies for each database are available in Table 3, Table 4 and Table 5.

### 2.2. Eligibility Criteria

Studies that met all of the following criteria were included:

- Quantitative studies, including population surveys, analyses of screening coverage or adherence data, and observational studies (such as cross-sectional, case–control, or cohort studies).
- Qualitative studies, including semi-structured interviews, focus groups, ethnographies, and other approaches that explore women’s perceptions, experiences, and attributed meanings.
- Mixed-methods studies combine quantitative and qualitative approaches.
- Studies conducted on women of any age group, permanently residing in the Amazon region (urban and/or rural areas of the countries that make up the Amazon Basin).
- Studies conducted in any country or administrative subdivision that is part of the Amazon region, according to a widely accepted geopolitical and/or ecological definition.
- Studies addressing cervical cancer screening (Pap smear, HPV-DNA testing, cytopathology, etc.) and/or treatment.
- Studies describing at least one barrier to accessing cervical cancer screening or treatment, including geographical, socioeconomic, cultural, linguistic, organizational, or health system-related barriers.
- Original articles published in peer-reviewed journals, with no language limitation.
- Articles with no temporal limitation.

All references identified through the database searches were transferred to Rayyan (https://rayyan.ai/; accessed on 7 November 2025), a widely recognized platform that supports the management of systematic, scoping, and narrative reviews. The software was used to organize and structure the screening workflow, enabling blinded and independent evaluation of titles and abstracts according to the predefined inclusion criteria. The platform automatically detected potential duplicate records; these were reviewed by the research team and removed to avoid redundancy in the dataset.

Screening and selection of studies were conducted independently by two reviewers, who evaluated titles, abstracts, and full texts using predefined eligibility criteria. An agreement between the two reviewers was required for a study to be included. Any discrepancies were resolved through discussion or, when necessary, adjudicated by a third reviewer until consensus was achieved. This approach ensured methodological rigor, minimized selection bias, and maintained consistency in decision-making.

Data extraction was likewise performed independently by two reviewers using a standardized extraction form. Extracted information included study characteristics, population details, screening and treatment barriers, and key outcomes. Divergences between the two extracted datasets were reconciled through iterative comparison and consensus. When essential information was missing or unclear, the study authors were contacted to clarify methodological details or provide additional data.

### 2.3. Exclusion Criteria

Studies presenting any of the following characteristics were excluded:

- Narrative reviews, systematic reviews, or meta-analyses (these will only be consulted for manual search of additional references).
- Case reports or case series, letters to the editor, editorials, comments, conference abstracts, and gray literature that is not peer-reviewed.
- Studies were conducted with participants who are not permanent residents of the Amazon Region.
- Studies conducted exclusively with health professionals, managers, and policymakers, without collecting primary data from the target female population.
- Studies that do not present results related to barriers to accessing cervical cancer screening/diagnosis or treatment.
- Research conducted in regions not included in the geopolitical or ecological definition of the Amazon.

### 2.4. Risk of Bias Evaluation

Risk of bias was assessed using the Joanna Briggs Institute (JBI) Critical Appraisal Checklist for Analytical Cross-Sectional Studies, the JBI Critical Appraisal Checklist for Qualitative Research, the Newcastle–Ottawa Scale (NOS), and the Mixed-Methods Appraisal Tool (MMAT). Two independent authors assessed the risk of bias across specific domains. The overall risk of bias for each study was similarly classified. The risk of bias assessment was not used as a criterion for study exclusion; instead, it was employed to ensure transparency and to inform the interpretation of the findings within the synthesis and discussion of the results.

### 2.5. Data Collection Process and Synthesis Methods

A narrative synthesis was conducted using an a priori-defined analytical framework. Studies were grouped according to the similarity of reported outcomes, focusing on six predefined domains: geographic, socioeconomic, cultural, communication, organizational, and health system-related barriers, which reflect analytical categories frequently described in the literature on barriers to healthcare access [11,13,17,18,22]. Within each domain, findings were examined across different study designs and populations. This process combined a descriptive comparison of quantitative measures with a thematic integration of qualitative conclusions to identify consistent patterns across the Amazonian context.

To support the synthesis, a comprehensive table was developed to summarize key characteristics of each included study, including authorship and year, country, study setting, Amazonian context, objectives, design, data collection methods, population characteristics, type of service addressed (screening, treatment, or both), categories of barriers and facilitators, reported limitations, and principal conclusions.

### 2.6. Data Analysis

To increase confidence in the synthesized results, sensitivity analyses were conducted by excluding studies of lower methodological quality. Confidence in the body of evidence was assessed qualitatively, prioritizing consistency across studies and the potential for reporting bias. Owing to the limited number of studies from the Amazon Region and the heterogeneity in study designs and outcome measures, a meta-analysis was not feasible. Accordingly, results were examined using a descriptive analytical approach.

## 3. Results

### 3.1. Literature Search

The systematic search for studies identified a total of 57 records. Of these, 54 were retrieved through database searches (PubMed, *n* = 23; Web of Science, *n* = 23; and SciELO, *n* = 8), and three were identified through citation searching. Before screening, 23 duplicate records were removed, resulting in a total of 34 unique records eligible for the next phase.

**Figure 1:**
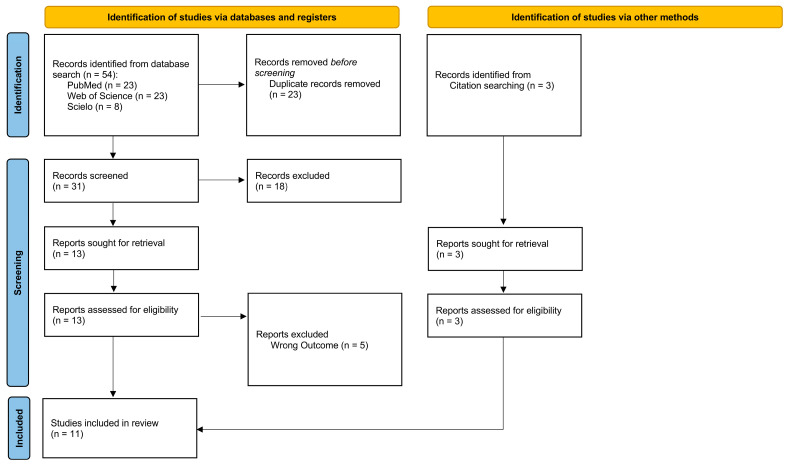
PRISMA flow diagram, the literature method search: n, the number of articles.

Following title and abstract screening, 18 records were excluded based on the predefined inclusion and exclusion criteria. Reports sought for retrieval totaled 16 (13 from the main search and three from other methods). Subsequently, 16 reports were assessed for eligibility. During this assessment, five reports were excluded because they addressed the wrong outcome. The systematic review ultimately included 11 studies. The PRISMA flow diagram is summarized in Figure 1.

### 3.2. Bias Risk Evaluation

To assess the risk of bias and ensure the methodological quality of the included studies, each study was evaluated using the Joanna Briggs Institute (JBI) Critical Appraisal Checklist for Analytical Cross-Sectional Studies, JBI Critical Appraisal Checklist for Qualitative Research, Newcastle–Ottawa Scale (NOS), and Mixed-Methods Appraisal Tool (MMAT), according to the design of each study. Discrepancies in scoring between reviewers were addressed and resolved through consensus-based discussion. A summary of the risk of bias assessment results is presented in Table 6, Table 7, Table 8 and Table 9.

### 3.3. Studies Characteristics

A total of 11 studies were included in this systematic review. All research was conducted within the Amazonian context, reflecting the region’s geographical and sociocultural diversity. The included studies employed diverse methodological approaches, including qualitative, quantitative, and mixed-methods designs. Study populations primarily comprised women from indigenous or rural communities, as well as healthcare professionals involved in cervical cancer screening and care. Table 10 provides a concise overview of the main characteristics of the included studies, while the complete extracted data for all variables are available in Supplementary Table S2.

Seven studies were conducted in Brazil, two in Colombia, and two in Peru. Six studies were cross-sectional, three were qualitative, one employed a retrospective cohort design, and one used a mixed-methods approach. Overall, the available evidence on cervical cancer screening and treatment in the region remains predominantly observational and descriptive. The studies varied widely in sample size, ranging from small qualitative investigations to large population-based surveys with representative household samples.

The studies evaluated women from diverse age groups, ethnic backgrounds, socioeconomic conditions, and geographic locations, including urban, peri-urban, rural, riverine populations, as well as socially isolated areas. Data collection methods included structured questionnaires, medical record abstraction, semi-structured interviews, and focus groups. Nine studies assessed barriers to screening, whereas two studies evaluated obstacles to treatment.

Rather than treating the Amazon Region as a homogeneous setting, the synthesis considered country-level and subregional differences across the included studies. Variations between Brazil, Colombia, and Peru, as well as distinctions among urban, peri-urban, rural, riverine, and socially isolated contexts, were considered during data extraction and interpretation. These territorial characteristics were analyzed as structural determinants that shape access to cervical cancer screening and treatment, and were integrated into the narrative synthesis.

#### 3.3.1. Description of the Studied Populations and Cultural Backgrounds

The studies included in this systematic review encompassed highly heterogeneous female populations in sociocultural, territorial, and economic terms, reflecting the complexity of the Amazonian context. The investigations involved women residing in regional capitals (such as Rio Branco, Manaus, and Iquitos), peripheral urban areas, rural zones, riverine communities, and Indigenous territories, which are often accessible only by river or air.

In several studies, participants belonged to Indigenous groups or traditional populations, including riverine communities and populations of Indigenous or mixed heritage, whose ways of life, social organization, and relationship with the territory are closely linked to rivers and the forest. A predominance of low educational attainment, engagement in subsistence activities (family farming, fishing, extractivism, or domestic work), low income, and near-exclusive reliance on public health services was observed, particularly on Brazil’s Unified Health System and on local public health networks across different Amazonian countries.

In these communities, traditional care practices, the use of natural medicine, and the intergenerational transmission of knowledge—often learned from mothers and grandmothers—coexist with biomedical services and shape decisions related to health-seeking behavior. In some Indigenous groups, cultural norms associated with shame, bodily modesty, exposure of intimacy, and specific moral codes (such as the concepts of *aurra* and *yaitan*) were reported in relation to women’s perceptions of gynecological examinations.

Linguistic diversity also emerged as a relevant factor, with some women speaking exclusively Indigenous languages and relying on cultural mediators or translators to understand health-related guidance. Conversely, studies that involved the active participation of community leaders, community health workers, and culturally adapted communication strategies—including the use of local languages, visual materials, and campaigns conducted within the communities themselves—reported greater acceptance of screening, suggesting that alignment between health service organizations and local cultural contexts is a key determinant in positively influencing screening adherence behaviors.

**Table 10:** Summary Characteristics of the Included Studies.

Study, First Author, Country, Year Published	Amazon Context	Design of the Study	Population/Participants	Conclusions
Study 1 Borges MF Brazil, 2012 [24]	Urban areas of Rio Branco, a city in the Brazilian Amazon, marked by geographic scattering, socioeconomic vulnerabilities and limited access to and research on cervical cancer screening coverage.	Cross-sectional study	772 women, aged between 18 and 69 years old	Statistically significant prevalence rates for absence of screening were found in women 18–24 and 60–69 years of age, single, and with low income and low schooling, highlighting the need for greater intervention in the group of women most vulnerable to cervical cancer incidence and mortality.
Study 2 Collins, J.H Peru, 2019 [25]	Peru’s largest and northernmost region, Loreto, is covered almost entirely by the Amazon floodplains. The port city of Iquitos, the region’s capital, is one of the least accessible cities in the world	Cross-sectional study	121 women, aged above 15 years	With a focus on cervical cancer, our findings show women have limited knowledge of behavioural risk factors that may contribute to development of the disease and are largely unaware of the need to undergo regular screening or to seek medical treatment for suspected cases. Barriers to undertaking screening are extensive, driven mainly by fear of the screening process and an identified lack of accessible services. Over 80% of respondents highlighted the need for accessible screening services within their communities.
Study 3 Fonseca, A.J Brazil, 2015 [26]	Native women that differ by lifestyle and interaction with western society. Yanomami women are isolated deep in the Amazon with a hunter/gatherer lifestyle.	Cross-sectional study	664 women, between 12 and 92 years old	Isolated endogenous Yanomami women were more likely to be HPV+ and rates increased with age. Study of HPV in isolated hunter-gather peoples suggests that long-term persistence is a characteristic of prehistoric humans and patterns reflecting decreased prevalence with age in western society represents recent change. These studies have implications for cervical cancer prevention and viral-host relationships.
Study 4 González, A. Colombia, 2022 [27]	The Colombian Amazon is one of the country’s regions having the greatest ethnic and ecosystem variety as it borders Peru and Brazil and has high population mobility owing to its rivers’ navigability. Entering and travelling within this territory is difficult; there is little access to medical care and close to 40% of the population (mainly those living in dispersed rural and/or populated urban centres) live in poverty.	Qualitative and quantitative research	309 women, aged ≥ 18-year-olds or older, having an active sexual life, and having resided in the target community for at least one year	This study’s findings suggested the need for including novel strategies in screening programmes which will promote CC promotion and prevention activities going beyond hospital outpatient attendance to reach the most remote or widely scattered communities, having the same guarantees regarding access, opportunity and quality. Including education-related activities and stimulating the population’s awareness regarding knowledge about CC prevention could be one of the main tools for furthering the impact of attendance at and compliance with P&P programmes.
Study 5 Mariño, J.M. Brazil, 2025 [28]	The municipality of Coari is located in the central region of the state of Amazonas, along the middle stretch of the Solimões River. It is 363 km from the capital, Manaus, and can be accessed by boat (river route) or aircraft (air route).	Qualitative/The study report was prepared in compliance with the COnsolidated criteria for REporting Qualitative studies	37 women residing in the city of Coari (urban and riverside areas), aged between 18 and 64 years old and registered with the Family Health Strategy of the municipality of Coari (PHCU Enedino Monteiro—Pera and PHCU Enedino Monteiro—Ribeirinha). Women with a previous diagnosis of CC and a history of hysterectomy were excluded.	The main barriers affecting screening adherence rates were institutional, individual and psycho-emotional aspects. Structural actions related to health service management and educational actions aimed at prevention and health promotion may improve the effectiveness of screening programs.
Study 6 Morse, R.M. Peru, 2023 [29]	This study took place in the MRIS (Micro Red Iquitos-Sur) network, the largest public health network of Loreto, located in the Peruvian Amazon.	Qualitative	20 screen-positive women who were referred for follow-up care but for whom evidence of follow-up was not found	The challenges elucidated demonstrate the complexity of implementing a successful cervical cancer prevention program and indicate a need for any such program to consider the perspectives of women to improve follow-up after a positive screening test.
Study 7 Prado, P.R. Brazil, 2014 [30]	Rio Branco, a city in the Brazilian Amazon	Retrospective cohort	237 women had precancerous lesions and underwent treatment, constituting the studied cohort	These findings reinforce the need to implement socio-educational interventions that address cervical cancer risk factors in women from Rio Branco, including lectures and other educational activities that emphasize the influence of the number of pregnancies, age of first sexual intercourse, having a stable sexual partner, smoking, and preventive exams.
Study 8 Sarmiento-Medina, M.I. Colombia, 2024 [31]	The Paujil reserve is situated in the Department of Guainía, within the Colombian Amazon, is home to a significant indigenous population comprising various ethnicities and origins. While there is no precise record of the population count, estimates from 2018, when the project commenced, suggested around 4000 individuals from 12 different ethnic groups.	Qualitative	34 women aged between 19 and 50 years.	Understanding the factors that influence access to screening is crucial for reducing inequalities in service delivery for indigenous women. The involvement of trained leaders who can identify these factors and motivate women can have a positive impact on the acceptance and guidance of cervical cancer prevention programs.
Study 9 Silva, D.C.B. Brazil, 2022 [32]	Carried out in 38 rural riverside locations along the Rio Negro River from the rural area of Manaus to the municipality of Novo Airão, Amazonas, Brazil, covered by a fluvial primary healthcare team.	Cross-sectional	221 women aged 18–59 years, and women under the age of 18 who had children under the age of two years or were pregnant.	Although a good performance of the fluvial health teams was identified, with a satisfactory coverage of cervical cancer screening in the studied population, the findings showed that there are barriers for women in rural riverside locations to access the screening test, including organizational barriers. The results also suggest that healthcare teams face difficulties in adequately managed care, which was evidenced by the possible delays in performing the test, and the significant number of unsatisfactory samples. Furthermore, the findings reinforce the importance of regular health promotion actions, which can increase the ties between the women and the health teams.
Study 10 Souza, L.R. Brazil, 2025 [33]	State of Acre, in the Western Brazilian Amazon	Cross-sectional	403 women with cervical cancer treated from 2012 to 2017 in all hospital units authorized to provide oncological care in the city of Rio Branco, Acre. Exclusion criteria were women whose treatment protocol was defined and implemented outside Rio Branco, who presented tumors other than epithelial, those who had neoplasms in a second primary site, pregnant women who waited until the end of pregnancy to start treatment, and those at stage IVB because the treatment was palliative and not curative.	Delay in cervical cancer treatment initiation in Acre was shorter than in other regions of the country. Age > 40 years, waiting >30 days for a specialist consultation were positively associated with delay, while advanced stages were inversely associated.
Study 11 Torres, K.L. Brazil, 2021 [34]	Manaus is the capital city of Amazonas State in northern Brazil, with a total area of 11,401 km^2^, most of which is rural (96.3%).	Cross-sectional	72,230 screening cytology tests were collected among women aged 25–64.	in order to reduce incidence and mortality due to cervical cancer in Manaus, Amazonia region, and high-burden cities world-wide, public health system should assure high vaccination coverage through school-based or similar programs. Until the first cohort of vaccinated girls age past the peak of cervical cancer, secondary prevention will be needed to avert early deaths. For screening programs to be cost-effective, novel risk-based organized screening programs should use adaptable strategies to confront social determinants of health and reach high-risk women. Once identified, those at risk of developing cervical cancer must receive safe and effective treatment, with minimal hurdles for women and their providers.

#### 3.3.2. Barriers

Across the included studies, barriers to cervical cancer screening and treatment were consistently associated with socioeconomic, cultural, organizational, communication, geographic, and health system-related determinants. After categorizing all reported barriers according to six predefined domains, the most frequently identified barriers were as follows.

#### 3.3.3. Health System-Related and Organizational Barriers

Organizational and health system limitations were consistently identified across the included studies. Difficulties related to appointment scheduling were reported in multiple studies. Long waiting times were also reported in more than one study, including instances of “long wait times” and cases in which patients waited more than 30 days for specialist consultation (PR = 2.94; 95% CI: 2.27–3.81; *p* < 0.05). Several studies cited a lack of health professionals and restricted access to providers or equipment, using terms such as “shortage of professionals,” “unavailability of providers,” and “equipment unavailability”.

Other organizational barriers reported in single studies included lack of access to services (53.6%, n = 60/112); lack of awareness of prior cytology results (3.3%, n = 22); lack of forms; delays in delivering test results; poor communication by healthcare staff; multi-step care processes; and limited knowledge of the healthcare unit responsible for providing the screening service (OR = 0.18; 95% CI: 0.04–0.97). Together, organizational and health system-related barriers accounted for the majority of all identified barriers.

#### 3.3.4. Socioeconomic Barriers

Multiple studies reported associations between socioeconomic characteristics and reduced adherence to cervical cancer screening. Younger age (<25 years) was associated with an increased risk of non-adherence (Adjusted PR = 3.19; 95% CI: 2.23–4.57), and older age (60–69 years) showed a similar pattern (Adjusted PR = 2.05; 95% CI: 1.45–2.88). Low income (≤1 minimum wage) was associated with non-adherence (Adjusted PR = 1.47; 95% CI: 1.13–1.92), and low educational level (≤elementary schooling) was also linked (Adjusted PR = 1.74; 95% CI: 1.37–2.20). Being single, divorced, or widowed was reported as a barrier (Adjusted PR = 1.77; 95% CI: 1.22–2.58), and women who were not in a stable relationship showed reduced adherence (likelihood-ratio *p* = 0.025).

Additional socioeconomic findings were reported in individual studies. Out-of-pocket payments, travel costs, and financial difficulties related to missing days of work were identified as relevant barriers. Multiparity was associated with lower screening uptake (OR = 0.76; 95% CI: 0.64–0.90), and having more than five pregnancies was also associated with reduced participation (52.9%; likelihood-ratio *p* = 0.005). Engagement in domestic activities was associated with a reduced likelihood of involvement in screening (OR = 0.31; 95% CI: 0.11–0.89). Age greater than 40 years was additionally reported as a barrier (PR = 1.75; 95% CI: 1.26–2.42; *p* < 0.05). This category of barriers was highly prevalent, particularly in studies conducted in rural and riverine areas of the Amazon Region.

#### 3.3.5. Cultural Barriers

Fear related to the screening process was the most frequently reported cultural barrier. This finding was reported in two studies, including one in which 70.8% of participants (n = 80/113) reported fear of the examination. Limited knowledge about cervical cancer was also identified. In one study, 32.7% of women (n = 34/116) had heard of cervical cancer, 22.0% (n = 25/116) were able to explain what cervical cancer is, and only 6.0% (n = 7/116) correctly identified Pap smears as a preventive measure.

Additional cultural barriers were reported in individual studies. These included misinformation, feelings of shame, restrictions imposed by husbands, difficulty managing time, fatalistic beliefs, lack of awareness of HPV or cervical cancer screening, limited understanding of follow-up and treatment procedures, and distrust toward health services. No quantitative measures were provided for these individual barriers.

Cultural dimensions were informed primarily by qualitative and mixed-methods studies that explored women’s perceptions, beliefs, and experiences related to cervical cancer prevention and care. These studies provided contextual insight into fear of the disease and screening procedures, feelings of shame, gender norms, and family-related dynamics among Indigenous, riverine (“ribeirinho”), and quilombola communities. Overall, the findings suggest that cultural beliefs and varying levels of health literacy may influence how information about screening and treatment is perceived and acted upon across multiethnic Amazonian settings.

#### 3.3.6. Geographical Barriers

Difficulties related to geographic access were described in more than one study. The need to leave riverine communities to receive care was reported, as was reduced screening adherence among women living in rural areas (adjusted OR = 0.43, 95% CI: 0.24–0.79, *p* = 0.006). Spatial factors within urban areas were also identified. One study found an inverse correlation between living in larger neighborhoods and screening uptake (Pearson’s r = −0.39; *p* = 0.005), and another reported lower participation among women living in Southern and Western zones, which were described as having fewer Basic Health Units per capita (*p* < 0.0001). No additional quantitative geographic measures were reported in the remaining studies.

#### 3.3.7. Communication Barriers

None of the included studies reported communication-related barriers. Although linguistic challenges are widely recognized as potential obstacles in multiethnic and Indigenous regions of the Amazon, none of the reviewed publications identified language-related difficulties as barriers to cervical cancer screening or follow-up.

#### 3.3.8. Facilitators to Screening and Treatment of Cervical Cancer in the Amazon Region

Across the included studies, several factors were identified as facilitators that improved women’s participation in screening programs, adherence to follow-up, and completion of treatment. A recurrent enabling factor was women’s positive attitudes and willingness to engage in preventive care, with many reporting motivation to undergo screening and to continue along the care continuum when services were accessible. Participation increased when invitations were active, when screening programs were organized and offered within community settings, and when health campaigns or public awareness strategies were implemented.

Support networks were instrumental, as support from family members, partners and peers was associated with higher attendance and improved continuity of care. Being in a stable relationship was protective, facilitating access and adherence, as reported in one study. The presence and active role of community health workers were repeatedly described as essential, particularly in rural and remote territories, as they improved communication, mobilization, and trust among local populations. In addition, service integration and the existence of clear care pathways promoted progression along the treatment continuum, with high motivation observed among women who had already entered the system.

Some studies also highlighted that advanced clinical staging acted as an unplanned facilitator for treatment initiation, likely driven by the perception of disease severity and the urgent need for care. Joint breast and cervical cancer awareness campaigns were likewise associated with increased screening uptake when implemented.

Overall, the identified facilitators highlight the importance of community-based strategies, social support, educational initiatives, and structured health service organization in strengthening screening coverage and reducing loss to follow-up in the cervical cancer care pathway.

#### 3.3.9. Limitations

Across the included studies, the most frequently reported limitations were related to sampling and study design. Cross-sectional designs were commonly noted as limiting the ability to infer temporality or causality between risk factors, screening behaviors, and outcomes. Several studies have mentioned small or non-representative samples, particularly due to challenges in accessing remote or riverine Amazonian communities, which can lead to potential selection bias.

Information bias was recurrent, primarily because many studies relied on self-reported data, which introduced recall bias, imprecise reporting of medical history, and difficulties related to calendar or date accuracy, particularly among Indigenous populations. The potential for social desirability bias was also evident, mainly in qualitative studies and focus groups, as some participants may have felt compelled to provide socially acceptable answers or to avoid expressing dissenting views.

In some studies, language and sampling limitations were noted, such as the exclusion of women who were not fluent in Spanish or recruitment restricted to health service users, which may have led to the underrepresentation of women who had never undergone screening. Additionally, retrospective and administrative data analyses were affected by incomplete clinical records, a lack of information on follow-up activities, or missing socioeconomic and geographic data. In a few cases, the authors did not report any study limitations.

Taken together, these issues raise concerns regarding selection bias, recall bias, measurement bias, and information bias. Such biases limit the generalizability of the available evidence and may affect the reliability of the findings in reflecting cervical cancer screening and treatment in Amazonian populations.

Despite these contributions, the predominance of cross-sectional data limits the ability to infer causality. To provide more robust evidence, future research could transition toward longitudinal or prospective designs capable of capturing temporal changes. Furthermore, the use of larger and more diverse representative samples, combined with mixed-methods approaches, would help overcome current sample size constraints and offer a more nuanced understanding of the contextual factors involved.

## 4. Discussion

This review identified multiple interconnected barriers to cervical cancer screening and treatment among women in the Amazon Region. These barriers are broadly consistent with global trends reported in the cervical cancer screening literature, particularly in low- and middle-income settings, where limited health literacy, socioeconomic disadvantage, geographic inaccessibility, and organizational constraints are commonly described. However, findings from the Amazon Region highlight how specific territorial characteristics, including vast geographic distances, reliance on river-based transportation, and fragmented healthcare delivery networks intensify these barriers. While similar challenges have been reported worldwide, the Amazonian context underscores the extent to which geographic and structural determinants interact to shape access to cervical cancer prevention and care.

A recent meta-analysis showed that women with higher levels of education, stable partnerships, public insurance, and greater household income were much more likely to undergo cervical cancer screening [35]. Similar findings have been reported in other settings, indicating that socioeconomic inequalities strongly influence women’s participation in preventive care, timely diagnosis, and sustained engagement along the care continuum [36,37,38,39]. The present findings further demonstrate how social determinants of health are intrinsically linked to cervical cancer prevention and how economic hardship often translates into unequal protection against the disease.

Geographic accessibility emerged as a major barrier across the included studies. Across multiple Amazonian regions, transportation difficulties, precarious road and river networks, long travel distances to referral centers, and limited availability of oncology services contribute to delays in diagnosis and treatment [11,32,33].

These geographic challenges are particularly pronounced in vast rural areas, where river routes often represent the only feasible means of transportation. In settings with low density of health facilities, delayed access to cytology reading, colposcopy, and biopsy results intensifies clinical risk and reduces opportunities for early detection. Similar challenges have been described in isolated and rural contexts outside the Amazon, particularly in Sub-Saharan Africa and Southeast Asia, suggesting that physical access remains a global concern for cervical cancer control [40,41].

Notably, some studies indicate that targeted governmental or non-governmental interventions can mitigate disadvantage in specific contexts [41,42]. Community-based educational initiatives, strengthened primary care teams, and sustained outreach campaigns have been shown to improve adherence over time [41,43]. In geographically dispersed regions, mobile health units, river-based clinics, and HPV self-collection represent feasible alternatives when facility-based screening models fail to reach high-need populations [41,42]. These findings suggest that prevention programs are more effective when they prioritize modes of care delivery rather than focusing solely on individual responsibility.

Support from family and community relationships plays an important role. Marital status consistently emerged as a significant factor, with married women or those in stable relationships being more likely to adhere to preventive care [36,43]. Educational level is also directly associated with screening uptake [35,44], particularly because health literacy affects the interpretation of risk, fear of cancer, and willingness to undergo pelvic examinations. In this review, women with limited formal schooling were less likely to undergo screening or seek timely treatment. Low health literacy and limited knowledge regarding cervical cancer prevention were repeatedly described as primary barriers to screening uptake [39,45].

Qualitative studies report that fear, embarrassment, misconceptions about cancer risk, and low perceived susceptibility remain significant barriers, particularly in rural and riverine communities. Educational strategies in the Amazon should address language diversity, value traditional knowledge, and engage trusted local actors, including community leaders, women’s groups, and faith-based organizations. Evidence indicates that culturally appropriate communication and participatory planning can improve screening uptake. Taken together, these findings highlight that effective interventions should emerge from local relationships and community contexts, rather than relying solely on information transfer [46].

While ethnicity did not appear to directly influence screening participation in this review, structural inequalities experienced by minority groups remain a barrier to access preventative care [37]. Geographic isolation and the lack of culturally adapted services disproportionately affect Indigenous, “quilombola”, and riverine women.

Treatment barriers included poverty, shortages of specialized health professional staff, and delays in referral to diagnostic facilities, which reduced opportunities for early intervention [47]. In the Amazon, delays between abnormal cytology findings and definitive treatment often result in diagnoses at advanced stages, poorer outcomes, and avoidable mortality [39,47]. These findings are consistent with evidence from the literature showing that social vulnerability is commonly associated with delayed entry into cancer care, regardless of the existence of national screening policies [48].

These issues also have significant ethical implications. From a bioethical perspective, principles of justice and equity support prioritizing regions and populations with greater needs. Structural vulnerability requires targeted interventions that address cultural, territorial, and historical characteristics. HPV self-collection kits, mobile screening campaigns, telemedicine-enabled follow-up, strengthened primary care networks, and culturally adapted health education materials may be effective in this context [42,46]. Participatory planning with community leaders could enhance trust and reduce historical tensions with healthcare institutions. At the same time, evidence from high-income settings also indicates that social gradients in screening participation persist, even when overall coverage is high [49].

This review has some limitations, as the heterogeneity across study designs, populations, and measurements of screening practices complicates direct comparisons. Few studies have addressed the specific regional characteristics of the Amazon region, and qualitative data remain limited, despite their importance for understanding lived experiences and the ethical dimensions of care.

Future studies should examine how culturally responsive interventions influence both screening and treatment outcomes. Incorporating community-led perspectives, particularly from Indigenous and riverine communities, will be crucial for reducing disparities and developing interventions that are both effective and ethically grounded.

Upon reviewing the studies, it appears that clinical measures alone are insufficient; it is also necessary to consider the social and environmental conditions that influence women’s access to care in the Amazon Region. Efforts to reduce preventable deaths extend beyond expanding screening availability and must include building trust, enhancing service organization, and promoting collaboration among communities, healthcare professionals, and policy makers. Interventions that incorporate local knowledge and uphold cultural diversity tend to be both more effective and ethically defensible. Implementing these strategies demands time and sustained political engagement. However, the potential improvements in equity, health outcomes, and the well-being of the women most affected, render such sustained efforts both necessary and justified.

## 5. Conclusions

The findings of this systematic review demonstrate that organizational and health-system-related barriers were frequently documented across the included studies, with common challenges including appointment scheduling difficulties, prolonged wait times, restricted availability of health professionals and available equipment. Socioeconomic characteristics associated with reduced screening included younger and older age, low income, limited educational attainment, and financial barriers related to travel or missing work due to illness. Cultural barriers encompassed apprehension regarding screening and insufficient knowledge of cervical cancer and its preventive examinations. Geographic barriers were identified in rural and riverine contexts, including lower screening uptake among women living in areas with fewer basic health units.

Non-adherence to cervical cancer screening and treatment in the Amazon Region arises from a complex interaction of systemic, educational, and individual factors, including lack of provider-initiated testing, low cancer literacy, procedural discomfort, stigma, and perceived inaccessibility of services. From a bioethical standpoint, the ethical imperative is clear: women in the Amazon should not face preventable harm as a result of geography, poverty, or the absence of culturally appropriate cervical cancer screening and treatment.

Given the organizational, geographic, socioeconomic, and cultural barriers identified in this review, it is imperative to implement equity-focused actions for populations at risk for cervical cancer. These actions include the expansion of affordable HPV vaccination, early and culturally adapted screening programs, and timely treatment regardless of social or economic status. Sustained outreach campaigns and community-based initiatives can improve adherence over time, while locally delivered models—such as mobile health units, river-based clinics, and HPV self-collection—offer feasible alternatives in geographically dispersed settings. Modernizing access through digital information systems further supports continuity of care.

Based on the evidence synthesized in this review, specific recommendations can be drawn for policymakers and healthcare providers. Policymakers should prioritize strengthening primary healthcare infrastructure in remote and underserved Amazonian areas by reducing organizational barriers, such as long waiting times and limited appointment availability. This can be achieved by expanding access to affordable HPV vaccination and ensuring decentralized, timely screening and treatment services. Healthcare providers can contribute by promoting provider-initiated screening, delivering culturally sensitive and linguistically appropriate communication, and supporting patient-centered education strategies that aim to improve cervical cancer literacy and reduce fear and stigma. Together, these actions may help translate evidence into practice and reduce preventable inequities in cervical cancer prevention and care in the Amazon region.

Lastly, the included studies revealed significant methodological and contextual limitations that should be taken into account when interpreting the evidence. Common issues included small and non-representative samples, predominance of cross-sectional or retrospective designs, and frequent information and recall biases. Future research should consider adopting more robust study designs, improving data documentation, and ensuring greater inclusion of Indigenous, riverine, and linguistically diverse populations to strengthen the evidence base for cervical cancer prevention and treatment in the Amazon Region.

## Figures and Tables

**Table 1:** PICO Framework.

PICO Components	Description
P (Population)	Women, of any age, who permanently reside in the Amazon Region (including urban and rural areas of the region’s countries), with or without a diagnosis of cervical cancer, who have been the target of screening or treatment programs.
I (Intervention)	Services, programs, and policies aimed at cervical cancer screening (e.g., Pap smear, cytopathology, HPV-DNA testing) or treatment.
C (Comparison)	Not directly applicable, but we may consider comparisons between population subgroups (e.g., by race/ethnicity, income level, urban/rural area, country).
O (Outcome)	Barriers to access (geographical, socioeconomic, cultural, linguistic, organizational, and health system-related), including analysis of intersectionality and combined effects on screening behaviors and treatment adherence.

**Table 2:** SPIDER Framework.

SPIDER Components	Description
S (Sample)	Women of any age permanently residing in the Amazon Region of any country, encompassing diverse racial, ethnic, socioeconomic, and geographic groups.
PI (Phenomenon of Interest)	Experiences, perceptions, and obstacles related to accessing cervical cancer screening and treatment.
D (Design)	Qualitative studies (interviews, focus groups, ethnographies), quantitative studies (population surveys, observational studies), and mixed-methods designs.
E (Evaluation)	Identification, categorization, and analysis of the type and magnitude of barriers, as well as reported strategies to reduce or overcome them
R (Research type)	Qualitative, quantitative, and mixed-methods studies published in peer-reviewed journals.

**Table 3:** Query box for PubMed search.

PubMed
query #1 “Uterine Cervical Neoplasms”[Mesh] OR “cervical cancer”[tiab] OR “cervical neoplasm*”[tiab] OR “cervix cancer”[tiab] OR “cervix neoplasm*”[tiab]
query #2 “Mass Screening”[Mesh] OR “Early Detection of Cancer”[Mesh] OR “cancer early detection”[Mesh] OR “cancer early diagnosis”[Mesh] OR “Papanicolaou Test”[Mesh] OR “self testing”[MeSH Terms] OR “Colposcopy”[tiab] OR “HPV DNA Tests”[tiab] OR screen*[tiab] OR “Pap smear”[tiab] OR cytolog*[tiab] OR colposcop*[tiab] OR “HPV test*”[tiab] OR “visual inspection with acetic acid”[tiab] OR VIA[tiab] OR VILI[tiab] OR “self-sampl*”[tiab] OR self-collect*[tiab] OR treatment[tiab] OR therap*[tiab] OR surgery[tiab] OR hysterectom*[tiab] OR conization[tiab] OR LEEP[tiab] OR radiotherap*[tiab] OR brachytherap*[tiab] OR chemoradiotherap*[tiab]
query #3 “Health Services Accessibility”[Mesh] OR “Delivery of Health Care”[Mesh] OR “Health Equity”[Mesh] OR “Rural Health Services”[Mesh] OR “Medically Underserved Area”[Mesh] OR “Transportation of Patients”[Mesh] OR “Patient Acceptance of Health Care”[Mesh] OR “Patient Compliance”[Mesh] OR “Healthcare Disparities”[tiab] OR barrier*[tiab] OR obstacle*[tiab] OR access*[tiab] OR accessib*[tiab] OR disparit*[tiab] OR inequ*[tiab] OR delay*[tiab] OR wait*[tiab] OR “time to treatment”[tiab] OR “time to diagnosis”[tiab] OR distance[tiab] OR transportation[tiab] OR travel[tiab] OR “out-of-pocket”[tiab] OR cost*[tiab] OR affordab*[tiab] OR availab*[tiab] OR acceptab*[tiab] OR uptake[tiab] OR coverage[tiab] OR participation[tiab] OR adherence[tiab] OR compliance[tiab] OR “loss to follow-up”[tiab] OR “missed appointment*”[tiab] OR “no-show*”[tiab] OR stigma[tiab] OR “health literacy”[tiab]
query #4 “women”[MeSH Terms] OR “female”[MeSH Terms] OR woman[tiab] OR Human* [tiab]
query #5 “Amazon Region”[tiab] OR “Amazon Basin”[tiab] OR Amazonia[tiab] OR Amazon[tiab] OR “Legal Amazon”[tiab] OR Amazonas[tiab]
query #6 query #1 AND query #2 AND query #3 AND query #4 AND query #5

**Table 4:** Query box for Web of Science search.

Web of Science
query #1 “Uterine Cervical Neoplasms” OR “cervical cancer” OR “cervical neoplasm*” OR “cervix cancer” OR “cervix neoplasm*”
query #2 “Mass Screening” OR “Early Detection of Cancer” OR “cancer early detection” OR “cancer early diagnosis” OR “Papanicolaou Test” OR “self testing” OR “Colposcopy” OR “HPV DNA Tests” OR screen* OR “Pap smear” OR cytolog* OR colposcop* OR “HPV test*” OR “visual inspection with acetic acid” OR VIA OR VILI OR “self-sampl*” OR “self-collect*” OR treatment OR therap* OR surgery OR hysterectom* OR conization OR LEEP OR radiotherap* OR brachytherap* OR chemoradiotherap*
query #3 “Health Services Accessibility” OR “Delivery of Health Care” OR “Health Equity” OR “Rural Health Services” OR “Medically Underserved Area” OR “Transportation of Patients” OR “Patient Acceptance of Health Care” OR “Patient Compliance” OR “Healthcare Disparities” OR barrier* OR obstacle* OR access* OR accessib* OR disparit* OR inequ* OR delay* OR wait* OR “time to treatment” OR “time to diagnosis” OR distance OR transportation OR travel OR “out-of-pocket” OR cost* OR affordab* OR availab* OR acceptab* OR uptake OR coverage OR participation OR adherence OR compliance OR “loss to follow-up” OR “missed appointment*” OR “no-show*” OR stigma OR “health literacy”
query #4 women OR female OR woman OR Human*
query #5 “Amazon Region” OR “Amazon Basin” OR Amazonia OR Amazon OR “Legal Amazon” OR Amazonas
query #6 query #1 AND query #2 AND query #3 AND query #4 AND query #5

**Table 5:** Query box for SciELO search.

SciELO
query #1 “Uterine Cervical Neoplasms” OR “cervical cancer” OR “cervical neoplasm*” OR “cervix cancer” OR “cervix neoplasm*” OR “câncer cervical” OR “câncer de colo de útero” OR “neoplasia cervical” OR “cáncer de cuello uterino”
query #2 “Mass Screening” OR “Early Detection of Cancer” OR “cancer early detection” OR “cancer early diagnosis” OR “Papanicolaou Test” OR “self testing” OR “Colposcopy” OR “HPV DNA Tests” OR screen* OR “Pap smear” OR cytolog* OR colposcop* OR “HPV test*” OR “visual inspection with acetic acid” OR VIA OR VILI OR “self-sampl*” OR “self-collect*” OR treatment OR therap* OR surgery OR hysterectom* OR conization OR LEEP OR radiotherap* OR brachytherap* OR chemoradiotherap* OR rastreamento OR diagnóstico precoce OR colposcopia OR tratamento OR cirurgia OR radioterapia
query #3 “Health Services Accessibility” OR “Delivery of Health Care” OR “Health Equity” OR “Rural Health Services” OR “Medically Underserved Area” OR “Transportation of Patients” OR “Patient Acceptance of Health Care” OR “Patient Compliance” OR “Healthcare Disparities” OR barrier* OR obstacle* OR access* OR accessib* OR disparit* OR inequ* OR delay* OR wait* OR “time to treatment” OR “time to diagnosis” OR distance OR transportation OR travel OR “out-of-pocket” OR cost* OR affordab* OR availab* OR acceptab* OR uptake OR coverage OR participation OR adherence OR compliance OR “loss to follow-up” OR “missed appointment*” OR “no-show*” OR stigma OR “health literacy” OR barreiras OR acesso OR desigualdades OR disparidade OR custo
query #4 women OR female OR woman OR Human* OR mulher OR mulheres OR feminino OR hembra
query #5 “Amazon Region” OR “Amazon Basin” OR Amazonia OR Amazon OR “Legal Amazon” OR Amazonas OR Amazônia
query #6
query #1 AND query #2 AND query #3 AND query #4 AND query #5

**Table 6:** Risk of Bias Assessment Using the JBI Critical Appraisal Checklist for Analytical Cross-Sectional Studies.

Reference	1	2	3	4	5	6	7	8	Overall Risk
Borges, 2012	Yes	Yes	Yes	No	Yes	Yes	Yes	Yes	Moderate
Collins, 2019	Yes	Yes	Yes	Yes	No	No	Yes	Yes	Moderate
Fonseca, 2015	Yes	Yes	Yes	Yes	Yes	No	Yes	Yes	Moderate
Silva, 2022	Yes	Yes	Yes	Yes	Yes	Yes	Yes	Yes	Low
Souza, 2025	Yes	Yes	Yes	Yes	Yes	No	Yes	Yes	Moderate
Torres, 2021	Yes	Yes	Yes	Yes	Yes	No	Yes	Yes	Moderate

Key: (1) Inclusion criteria clearly defined? (2) Subjects & setting described? (3) Exposure measured validly & reliably? (4) Objective standard criteria for outcome? (5) Confounding factors identified? (6) Strategies to deal with confounding? (7) Outcomes measured validly & reliably? (8) Appropriate statistical analysis?

**Table 7:** Risk of Bias Assessment Using the JBI Critical Appraisal Checklist for Qualitative Research.

Reference	1	2	3	4	5	6	7	8	9	10	Overall Risk
Mariño, 2025	Yes	Yes	Yes	Yes	Yes	No	No	Yes	Yes	Yes	Low
Morse, 2023	Yes	Yes	Yes	Yes	Yes	No	Yes	Yes	Yes	Yes	Low
Sarmiento-Medina, 2024	Yes	Yes	Yes	Yes	Yes	Yes	Yes	Yes	Yes	Yes	Low

Key: (1) Is there congruity between the stated philosophical perspective and the research methodology? (2) Is there congruity between the research methodology and the research question or objectives? (3) Is there congruity between the research methodology and the methods used to collect data? (4) Is there congruity between the research methodology and the representation and analysis of data? (5) Is there congruity between the research methodology and the interpretation of results? (6) Is there a statement locating the researcher culturally or theoretically? (7) Is the influence of the researcher on the research, and vice versa, addressed? (8) Are participants and their voices adequately represented? (9) Is the research ethical according to current criteria, and is there evidence of ethical approval? (10) Do the conclusions drawn flow from the analysis or interpretation of the data?

**Table 8:** Risk of Bias Assessment Using the Newcastle–Ottawa Scale (NOS).

Reference	S1	S2	S3	S4	C1	O1	O2	O3	Total	Overall Risk
Borges, 2012	1	NA	1	1	2	1	1	1	7	Low

Key: S, Selection; C, Comparability; O, Outcome. (S1) Representativeness of the Exposed Cohort, (S2) Selection of the Non-Exposed Cohort, (S3) Ascertainment of Exposure, (S4) Demonstration that Outcome of Interest was not Present at Start of Study, (C1) Comparability of Cohorts on the Basis of Design or Analysis, (O1) Assessment of Outcome, (O2) Was Follow-Up Long Enough for Outcomes to Occur and (O3) Adequacy of Follow-up of Cohorts.

**Table 9:** Risk of Bias Assessment Using the Mixed-Methods Appraisal Tool (MMAT).

Reference	QL1	QL2	QL3	QL4	NR1	NR2	NR3	NR4	MM1	Total	Overall Risk
González, 2022	Yes	Yes	Yes	Yes	Yes	Yes	Yes	Yes	Yes	9/9	Low

Key: QL, Qualitative; NR, Quantitative Non-Randomized; MM, Mixed-Methods. (QL1) The qualitative design is appropriate to answer the research question. (QL2) The data collection is appropriate to answer the research question. (QL3) The researcher’s perspective has been adequately addressed. (QL4) The coherence between data collection, analysis, and interpretation is adequate. (NR1) The sampling strategy is appropriate to address the research question. (NR2) The measurement of the exposure/intervention and the outcomes are appropriate. (NR3) The risk of nonresponse bias is adequately addressed. (NR4) Confounding has been accounted for in the design and/or analysis. (MM1) The integration of the quantitative and qualitative components is appropriate and complete.

## Data Availability

Data sharing is not applicable to this article, as no new data were created or analyzed in this study. All data supporting the findings of this review are derived from previously published studies and are included in the article and its Supplementary Materials.

## Supplementary Materials

The following supporting information can be downloaded at: https://www.mdpi.com/article/10.3390/jcm15031206/s1, Table S1: PRISMA 2020 Checklist; Table S2: Complete Extracted Data for All Variables from the Included Studies.

## Abbreviations

The following abbreviations are used in this manuscript:

CC	Cervical cancer
HPV	Human papillomavirus
JBI	Joanna Briggs Institute
NOS	Newcastle–Ottawa Scale
MMAT	Mixed-Methods Appraisal Tool
